# Effects of Dietary Rapeseed (*Brassica napus*), Hemp (*Cannabis sativa*) and Camelina (*Camelina sativa*) Seed Cakes Supplementation on Yolk and Albumen Colour and Nutritional Value of Yolk Lipids in Estonian Quail Eggs

**DOI:** 10.3390/ani12223110

**Published:** 2022-11-10

**Authors:** Violeta Razmaitė, Artūras Šiukščius, Raimondas Leikus

**Affiliations:** Animal Science Institute, Lithuanian University of Health Sciences, R. Žebenkos 12, LT-82317 Baisogala, Lithuania

**Keywords:** egg, quail, yolk, colour, fatty acids, lipid indices, cholesterol

## Abstract

**Simple Summary:**

Despite the regional preferences for quail eggs and meat, and the main production being found in Asian countries, the popularity of quail raising has increased and their production has been introduced in many other countries, including the Baltics. A wide distribution around the world encouraged genetic divergence among Japanese quail populations, the formation of different breeds and lines as well as their research. The Estonian quail, a breed developed in Estonia, has also spread in Lithuania. This study aimed to evaluate yolk and albumen colour, as well as the nutritional value of yolk lipids in Estonian quail eggs using diets supplemented with cakes from local oil corns (rapeseed, hemp and camelina seeds). The diet affected the colour as well as the cholesterol content and fatty acid composition of the yolk. All quail groups demonstrated the high lightness (L*) and yellowness (b*) of the yolk; however, the negative value of parameter a*, instead of being red, showed a greenish colour. The highest proportions of total saturated (SFAs) and polyunsaturated (PUFAs) fatty acids as well as the lowest monounsaturated fatty acids (MUFAs) were found in the yolk lipids of the quail which was fed a mixture supplemented with hemp cake. The lowest and most favourable n-6/n-3 ratio was demonstrated by the quail fed the diet supplemented with camelina. However, the lowest yolk cholesterol content was demonstrated by the quail fed the diet supplemented with rapeseed cake.

**Abstract:**

The aim of this study was to investigate the effects of supplementing commercial diets with rapeseed, hempseed and camelina seed cakes on the yolk and albumen colour and nutritional value of yolk lipids in Estonian quail eggs. A total of 585 eggs were used to evaluate egg weight, yolk and albumen pH and colour, proximate yolk composition, cholesterol content and fatty acid composition in the yolk lipids of three Estonian quail groups each fed a diet supplemented with 10% rapeseed, hemp or camelina seed cakes. The higher (*p* < 0.01 and *p* < 0.05, respectively) lightness (L*) of the yolk was detected in the group of quail fed the diet supplemented with rapeseed and camelina cakes compared to the hempseed addition. Meanwhile, the albumen lightness in the rapeseed group was higher (*p* < 0.01) in comparison to the camelina group. A lower (*p* < 0.001) yolk negative a* value was estimated in the hemp group than in the groups fed rapeseed and camelina seed cake supplements, while the rapeseed group demonstrated lower (*p* < 0.001) albumen yellowness (b*) and colour saturation (C) in comparison to the other groups. The highest and lowest proportions of total saturated (SFAs)and monounsaturated (MUFAs) fatty acids, respectively, were found in the yolk lipids of quails fed a mixture supplemented by hemp cake. A higher (*p* < 0.05) proportion of polyunsaturated fatty acids (PUFAs) was only found in the hemp group compared to the camelina group. The highest and lowest (*p* < 0.001) n-6/n-3 PUFA ratios were found in the yolk lipids of quail receiving mixtures supplemented with rapeseed and camelina cakes, respectively. The yolk of the quail from the rapeseed group showed more favourable lower and higher (*p* < 0.01 and *p* < 0.05, respectively) atherogenic index (AI) and hypocholesterolemic/hypercholesterolemic (h/H) ratio compared to the hemp and camelina groups. The lower yolk cholesterol content was found in the eggs of the quail fed the diet supplemented with rapeseed cake compared to the eggs of the quail which were fed the diets containing more n-3 PUFAs from the addition of hemp and camelina cakes (*p* < 0.05 and *p* < 0.01, respectively).

## 1. Introduction

The bird egg is a relevant source of nutrients, containing all of the proteins, lipids, vitamins, minerals and growth factors required by the developing embryo, as well as a number of other factors to protect against bacterial and viral infections [1,2]. Being a rich and balanced source of essential nutrients, eggs have a special place among different products [3]. On the other side, although eggs still have to face many years of nutritionist recommendations aiming at restricting egg consumption to limit the incidence of cardiovascular disease, many different studies have shown that there was no evidence of a correlation between dietary eggs and an increase in plasma total cholesterol [4]. Due to their nutrient profile, variety and low cost as food, eggs remain a food product of high nutritional quality for people of different age groups and are one of the most popular foods extensively consumed worldwide [4,5,6]. Moreover, there are no cultural restrictions for egg usage [7]. The nutritional value of quail eggs is much higher than that of other eggs and provide significantly more nutritional value than do other foods [6,8].

Currently, in Lithuania, the majority of consumed eggs are chicken eggs. Goose, duck and quail eggs are occasionally used as a gourmet food; however, quail eggs are becoming increasingly popular and more commonly consumed. As in other countries, the increase in quail production is explained by their potential for meat production, high egg production, commercial diversity and fast return on investment [9]. Moreover, the quail are known for their easy maintenance, short generations, ability to be kept in relatively large numbers in small facilities and potential use as a model animal for different works [10,11].

Colour is an important characteristic and selection criterion for egg choice by consumers because they believe that a well coloured egg yolk (golden yellow to orange) is associated with healthiness and quality and are makes for superior eggs in respect to taste and nutritional value [12,13,14,15]. As yolk colour is primarily determined by the content of pigmenting carotenoids present in the feed [15], studies in the field of quail nutrition have not focused only on specific topics such as nutritional protein, energy and alternative feeds [9,11,16,17,18], but also on pigment agents [9,16,19].

Rapeseed, hemp and camelina seeds, in contrast to the imported soybean and sunflower, are local oil corns, and cakes from these seeds obtained after pressing and removing the seed oil are available products in Lithuania.

Results have been published considering the effects of quail feeding using hempseed [20], hempseed oil [7,21] and meal [22], canola meal and seeds [23,24], rapeseed meal [25,26] and camelina meal [27]; however, the results on the use of these oilseed cakes for quail feeding are scarce. Moreover, the egg’s weight and quality characteristics are affected by different factors, including the genotype of avian species. The published results on egg characteristics of the Estonian quail breed, which was developed from the post-war Japanese quail (C. coturnix japonica) [28] and recognized in 1987 [29,30], are also scarce.

The aim of this study was to examine the effects of supplementing commercial diets with rapeseed, hempseed and camelina seed cakes on the colour of yolk and albumen, as well as on the nutritional value of the yolk lipids in Estonian quail eggs.

## 2. Materials and Methods

### 2.1. Experimental Material, Design and Diets

A total of 585 eggs as research material were obtained for the egg evaluation from 180 (60 in each of the three experimental groups) laying females of the Estonian quail (Coturnix coturnix japonica). From these eggs, in each group, 15 accessions were prepared for the observations of each trait. The eggs were collected and randomly allocated to 3 treatment groups of young quails aged 9.5 weeks after 4 weeks of the beginning of laying. On the fifth week, eggs were collected from each group to determine egg quality parameters (egg weight, yolk and albumen pH and colour, proximate yolk composition, cholesterol content and fatty acid composition in yolk lipids).

The quail were housed indoors in Cimuka Comfortplast cages (24 quail per cage) at 25–27 °C. The cages were fully equipped with feeders and nipple drinkers. Each bird was provided with 250 cm^2^ floor space. The quail were hatched out when the natural lighting was L16.1 h: D7.9 h, and started to lay at midsummer when the natural lighting reached L17.3 h: D6.7 h, while the eggs for evaluation were collected when the natural lighting decreased to L16.6 h: D7.4 h. During the nights, artificial 40 W lamplights were used.

The feeds were produced in the enterprise Adzida. With the aim of avoiding genetically modified (GM) ingredients, this enterprise does not use imported soybean. The composition of the feeds for the laying quail and the nutritive value of the feeds are presented in Table 1. These feeds and the water for all the groups were provided ad libitum.

Additionally, 1.5 g chalk was given per each quail daily. Supplementary data regarding premix “Calvet” composition can be found in Table S1. The composition of fatty acid of the fat in the feed mixtures for the laying quail is presented in Table 2.

### 2.2. pH and Colour Measurements

Each sample was taken from six eggs for pH and colour measurements of the yolks and albumen and a total of 15 composite samples were obtained for each group. Then, the samples of yolks and albumen were placed on separate Petri dishes and covered with a thin transparent elastic cling foil. The colour of the yolk and albumen was determined in the CIE L* a* b* and L* C h colour spaces system using Minolta CR-410 colorimeter (Konica Minolta, Osaka, Japan) equipped with a C illuminant and 2° standard observer calibrated to a standard white calibration plate (Y = 85.3, x = 0.3173, y = 0.3251). The L*, a*, b*, C and h parameters corresponded to the lightness (L*−100/+100, black/white), a* (−100/+100, green/red), b* (−100/+100, blue/yellow). The chroma or colour saturation (C) indicates a difference between the respective colourity and the grey colour, and the hue (h) indicates the specific angle of a particular colour shade.

After colour measurements, the samples were placed in test tubes and the pH of the yolk and albumen was measured using a digital portable FiveGo^TM^ pH meter F2 (Mettler Toledo GmbH, Schwarzenbach, Switzerland) equipped with a Mettler Toledo puncture-type pH electrode LoT406-M6-DXK-S7/25 with a Xerolyt polymer electrolyte and an electrode-protecting puncture knife after device calibration, as described in the operating instructions of the meter.

### 2.3. Proximate Composition of Egg Yolk

The dry matter content was determined [31] by drying samples in an oven at 105 °C until a constant weight was obtained (method No. 950.46B; AOAC, 1990). The crude protein content was determined by the Kjeldahl method using the Kjeltec system 1002 apparatus (Foss-Tecator AB, Höganäs, Sweden), and a conversion factor of 6.25 was used to convert total nitrogen into crude protein (method No. 981.10; AOAC, 1990). Crude fat was determined by the Soxhlet extraction method (method No. 960.39; AOAC, 1990). Ash was determined by incineration in a muffle furnace at 550 °C for 24 h (method No. 920.153; AOAC, 1990). The samples were analysed in duplicate for all analytes. The content of protein, fat and ash were expressed as the weight percentage of yolk dry matter.

The cholesterol content in yolk was determined according to the extraction method described by Zhang et al. [32] and followed by HPLC separation and analysis on Shimadzu10 A HPLC system (Shimadzu Corp., Kyoto, Japan). The data collection and evaluation were performed by using LC Solution (Shimadzu Corp., Kyoto, Japan) operating system. The analytical column was LiChrospher 100 RP-18e, 150 × 4.6 mm, 5 µm (Alltech Associates Inc., Columbia, MD, USA) with a guard column (LiChrospher 100 RP-18, 7.5 × 4.6 mm). The cholesterol content was expressed as mg/g of wet yolk weight.

### 2.4. Fatty Acid Profiles

The extraction of lipids for fatty acid analysis was performed with a mixture of two volumes of chloroform (Chromasolv Plus for HPLC containing 0.5–1.0% ethanol as a stabilizer) and one volume of methanol as described by Folch et al. [33]. Methylation of the samples was performed using sodium methoxide: 5 mL of 25 wt % solution in methanol was added to the sample and stirred. After 1 h, 7 mL of HCL, 6 mL of hexane and 2 mL of H_2_O were added. The top layer was transferred into a new test tube and evaporated. Fatty acid methyl esters were prepared according to the procedure described by Christopherson and Glass [34]. The FAMEs were analysed using a gas liquid chromatograph (GC-2010 SHIMADZU, Kyoto, Japan) fitted with a flame ionization detector. The separation of the methyl esters of the fatty acids was affected on the capillary column Rt 2560 (100 m × 0.25 mm × 0.2 μm; Restek, Bellefonte, PA, USA) by temperature programming from 160 °C to 230 °C. The temperatures of the injector and detector were held at 240 °C and 260 °C, respectively. The rate of flow of the carrier gas (nitrogen) through the column was 0.79 mL/min. The peaks were identified by comparison with the retention times of the standard fatty acid methyl esters “37 Component FAME Mix” and trans FAME MIX k 110 (Supelco, Bellefonte, PA, USA). The relative proportion of each fatty acid was expressed as the relative percentage of the sum of the total fatty acids using the “GC solution” software for Shimadzu gas chromatograph workstations.

### 2.5. Lipid Quality Indices

Lipid quality indices, i.e., atherogenic index AI = [(4 × C14:0) + C16:0]/[n-6 PUFA + n-3 PUFA+ MUFA and thrombogenic index TI = [C14:0 + C16:0 + C18:0]/[0.5 × MUFA + 0.5xn-6PUFA + 3 × n-3 PUFA + n-3 PUFA/n-6 PUFA] were calculated according to Ulbricht and Southgate [35]. The hypocholesterolemic/hypercholesterolemic h/H = [(C18:1n-9 + C18:1n-7 + C18:2n-6 + C18:3n-6 + C18:3n-3 + C20:3n-6 + C20:4n-6 + C20:5n-3 + C22:4n-6 + C22:5n-3 + C22:6n-3]/[C14:0 + C16:0] ratio was calculated according to Fernández et al. [36]. The peroxidisability index PI = [(C14:n-7 + C16:1n-9 + C16:1n-7 + C17:1n-9 + C18:1n-9t + C18:1n-9 + C18:1n-7 + C18:1n-7 + C20:1n-9) × 0.025] + [(C18:2n-6t,c + C18:2n-6) × 1] + [(C18:3n-3 + C18:3n-6 + C20:3n-6) × 2] + [(C20:4n-6 + C22:4n-6) × 4] + [(C20:5n-3 + C22:5n-3) × 6] + [C22:6 × 8] was determined according to Du et al. [37].

### 2.6. Statistical Analysis

The data were subjected to the analysis of variance in the general linear (GLM) procedure in IBM SPSS Statistics 27 (IBM, Armonk, NY, USA) with post hoc LSD tests to determine the significance of differences of EM means between the groups. The GLM model included the fixed factor of the feeding group (rapeseed, hemp or camelina seed cake addition). The differences were regarded as significant when *p* < 0.05.

## 3. Results and Discussion

### 3.1. Weight of Eggs Used for Evaluation

The weight of the eggs in the hempseed quail group was 1.2 g lower (*p* < 0.05) than that in the rapeseed group (Table 3). Tolik et al. [2] reviewed the mean weight of quail egg to be about 11 g, but in the present study the weight of the eggs was lower. The eggs were also smaller than reported by Tikk et al. [30] for the Estonian quail. However, our eggs were evaluated at the beginning of laying, when the eggs were smaller [30].

Similar egg weights have been reported by Dudusola [38] and Song et al. [39]. Despite the recognized high nutritional value of hempseeds [40,41,42] for humans and animals, there are references [43] indicating that it is more suitable to use the extract of complex bioactive substances in animal diets than to add hemp in such form as in the cake from hemp processing. Some authors have even reported that hemp oil negatively affected the performance of growing quail [21]. Despite the relatively small weight of the eggs, all our groups demonstrated large yolks. Although Ibrahim et al. [24] reported that canola seeds increase yolk size, in the present study the differences in the yolk weight between the groups were insignificant.

### 3.2. pH and Colour

The albumen pH of the eggs of the quail fed rapeseed cake was higher (*p* < 0.001 and *p* < 0.01, respectively) compared to the hemp and camelina cake supplementations in the feed mixtures (Table 4), while the yolk pH was not affected by feeding. The albumen pH value in the rapeseed group was similar to the findings of Silva et al. [9] who used corn- and sorghum-based feeds for the quail; however, the yolk pH in the present study was lower. Overall, both the yolk and albumen pH values in the present study were higher than those obtained by other authors using unknown feeds [44].

The higher (*p* < 0.01 and *p* < 0.05, respectively) lightness (L*) of the yolk was measured in the groups of quail fed the feed mixture supplemented with rapeseed and camelina cakes compared to the hempseed cake addition. A higher (*p* < 0.01) albumen lightness was found in the rapeseed group compared to the camelina group. In the present study, the yolk and albumen lightness of quail eggs was higher than those reported by other authors [22,44]. However, colour depends not only on the composition of the object but also on its illumination environment and the angles of illumination and viewing [45]. As there are large variations in the instruments used and in the use of illuminant and colour coordinates [46], the comparison can be considered relative. The negative values of the colour parameter a* indicate a greenish colour both for the yolk and albumen. A lower (*p* < 0.001) yolk negative a* value was estimated in the hemp group than in the groups offered the rapeseed and camelina seed cake supplements. A negative but lower a* value in the albumen of quail eggs was reported by other authors [44]. There were no significant differences in yolk yellowness (b*) between the groups, while the albumen yellowness and colour saturation (chroma, parameter C) in the rapeseed group were lower (*p* < 0.001) compared to the other groups. In the present study, yolk yellowness in all the groups was higher than that reported by Cudufar et al. [22] who used feeds containing different levels of hemp seed meal. Additionally, there were differences between the groups in the h parameter. The yolk in the hempseed group had a lower (*p* < 0.001) h value while the same parameter for albumen in the rapeseed group was higher (*p* < 0.001) compared to the other groups.

### 3.3. Proximate Composition of Yolk

The yolk of quail eggs from the hemp group had a lower (*p* < 0.01) content of dry matter compared to rapeseed and camelina groups and a lower (*p* < 0.01) content of protein than in the camelina group (Table 5), but there were no significant differences between the groups in the yolk lipid content. Although the lipid content detected in the yolk of Estonian quail eggs fed the diets containing cakes of oil corn was similar to those reported by different authors [2,38,39,47,48,49], the content of dry matter, including the content of protein, was lower.

Despite their high nutritive value, the consumption of eggs is often considered to be responsible for some health problems, such as heart diseases, due to a high cholesterol content [2]. Therefore, there have been efforts to look for ways to lower the cholesterol content in eggs. The authors compared the cholesterol content in quail and chicken as well as other species’ egg yolk and reported different results. In some studies [50], there was no significant difference in the cholesterol content between quail and chicken eggs; however, in other studies [6,42], the yolk of quail eggs showed a higher cholesterol content compared to chicken yolk or a lower cholesterol content compared to ostrich and turkey egg yolks [3]. In the present study, the lower (*p* < 0.05 and *p* < 0.01, respectively) yolk cholesterol content was demonstrated by the quail fed the diet supplemented with rapeseed cake compared to the quail which were fed the diets containing more n-3 PUFAs hemp and camelina cakes, and this is in contrast with Alagawany et al. [5], who reported that a higher level of PUFAs in quail diets reduces cholesterol content. However, the obtained values of the cholesterol content in all the groups varied in the range determined by other authors [3,50,51]. The diets supplemented with linseed and rich in n-3 PUFAs [51,52] only showed an insignificant decrease in the cholesterol content in the yolk; however, canola seeds the reduced LDL cholesterol in the quail’s blood [24].

### 3.4. Fatty Acid Composition

The highest (*p* < 0.01) proportion of total saturated fatty acids (SFAs), including individual myristic (C14:0) and palmitic (C16:0) fatty acids, was found in the yolk lipids of the quail fed the mixture supplemented with hemp cake (Table 6). The same quail group demonstrated lower (*p* < 0.01 and *p* < 0.05, respectively) proportions of total monounsaturated fatty acids (MUFAs) compared to the rapeseed and camelina groups. The highest proportion of MUFAs was determined by the highest proportions of the most abundant individual monounsaturated fatty acids, such as palmitoleic (C16:1n-9) and oleic (C18:1n-9) acids. The yolk lipids in the eggs of the hemp group showed a 20.1% relatively higher (*p* < 0.05) proportion of total polyunsaturated fatty acids (PUFAs) only between the hemp and camelina groups. Epidemiological studies in different countries indicate a decreased risk of cardiovascular disease (CVD) and inflammation with an increasing consumption of very long chain (VLC) n-3 PUFAs [53,54,55,56]. Within polyunsaturated fatty acids, EPA (C20:5n-3) and DHA (C22:6n-3) appear to be the most important n-3 PUFAs, although the roles for DPA (C22:5n-3) acid are also emerging [55,56]. Despite lower total PUFAs, the highest proportions of the most beneficial to human health α-linolenic ALA (C18:3n-3), DPA (C22:5n-3) and DHA (C22:6n-3) were found in the yolk of the camelina group eggs. Trace amounts of EPA (C20:5n-3) were detected only in the yolk of the hemp and camelina groups. VLC n-3 PUFAs such as EPA, DPA and DHA are metabolically related to one another, and they can be synthesized from plant-derived n-3 fatty acids [53,54,55]. The initial substrate for this pathway is an essential ALA (C18:3n-3) fatty acid in animals. However, the capacity of ALA conversion into VLC n-3 PUFAs in separate human groups is considered to be limited [53,54,55]; therefore, the accumulation of these fatty acids in food is very important. Higher proportions of very long chain n-3 PUFAs in the yolk lipids of quail eggs from the hemp and camelina groups are explained by the proportion of ALA in the feed mixtures used. The lipids in the feed mixtures containing hemp and camelina cakes had 24.9% and 3.5 times, respectively, more ALA than in the feed mixture with rapeseed cake supplementation. The obtained results are consistent with the findings of other authors who enriched quail diets with n-3 PUFAs using flaxseed addition [11,51,52], hempseed meal and oil [7,20,22].

The composition of the feed containing different oil corn cake additions also appeared to show the effect on the n-6/n-3 PUFAs ratio (Table 7). The highest and lowest (*p* < 0.001) n-6/n-3 PUFAs ratios were found in the yolk lipids of quail receiving mixtures supplemented with rapeseed and camelina cakes, respectively. Numerous studies [57,58,59] have reported about the n-6/n-3 PUFAs imbalance in the current Western diet. The Palaeolithic diet was described as being in perfect balance (1:1), while the latter is deeply unbalanced in favour of n-6 PUFAs (20:1) [56,57]. As Liput et al. [59] reported, it is possible that the most favourable ratio of n-6 to n-3 acids in the diet may vary depending on the genetic background of the PUFAs’ biosynthesis enzymes, age, gender and health condition.

However, the recommendations of Bellagio’s report on healthy nutrition indicated that the ratio (4:1) of n-6 PUFAs to n-3 PUFAs in the diet should be the goal [60]. It can be observed that the yolk lipids in all the quail groups did not meet this recommendation; however, the most favourable n-6/n-3 ratio was demonstrated by the quail fed camelina supplementation. An unfavourable n-6/n-3 PUFAs ratio was also reported by other authors [3,51,52]; however, some authors have also indicated a drastic reduction in this ratio with a flaxseed addition to quail diets [11,51,52]. Moreover, Usturoi et al. [61], in their publication, indicated a favourable (3.49) n-6/n-3 PUFAs ratio in the lipids of quail yolk as a good alternative to chicken eggs.

The differences in the fatty acid composition appeared to affect the lipid quality indices in the yolk. The yolk of quail from the rapeseed group showed a more favourable lower and higher (*p* < 0.01 and *p* < 0.05, respectively) atherogenic index (AI) and hypocholesterolemic/hypercholesterolemic (h/H) ratio compared to the hemp and camelina groups. The thrombogenic index (TI) was higher (*p* < 0.05) in the hemp cake group than in the other groups. A lower and more favourable peroxidisability index (PI; *p* < 0.05) was found for the yolk of eggs from the quail group fed rapeseed cake. Higher AI (0.52) and TI (1.11) indices for quail yolk were reported by Sinanoglou et al. [3].

## 4. Conclusions

The applied CIE L* a* b* and L*C h colour space system demonstrated high lightness and yellowness as well as a light greenish shading of the yolk in all the groups. The yolk of the quail fed the diet supplemented with rapeseed and camelina cakes showed the higher lightness and greenish colour compared to the hempseed supplementation group. There were no significant differences in yolk yellowness between the groups, while the albumen yellowness and colour saturation (chroma) in the rapeseed group were lower compared to the other groups.

The n-3 PUFAs increased in the diets supplemented with hemp, and camelina seed cakes decreased the n-6/n-3 PUFAs ratios in the yolk of quail eggs, and the most favourable n-6/n-3 ratio was demonstrated by the quail fed camelina supplementation. However, n-3 PUFAs-rich diets did not result in reduced cholesterol content. Moreover, the yolk of quail from the rapeseed group exhibited a more favourable lower and higher atherogenic index (AI) and hypocholesterolemic/hypercholesterolemic (h/H) ratio compared to the hemp and camelina groups. The obtained information showed that improvements in separate qualitative attributes does not indicate the same benefits in other characteristics. This information can be used to promote choice in quail feeds and production, as well as consumption. It also provides new insights for research into quail production, but further additional investigations on oil corn cakes and other factors on the quality of quail eggs are needed.

## Figures and Tables

**Table 1:** Composition of laying quail feed and its nutritive value.

Ingredients	Feeding Group		
	Rapeseed	Hemp	Camelina
Wheat,%	29.36	27.47	30.33
Barley, %	6.00	6.00	6.00
Maize, %	20.00	20.00	20.00
Peas, %	12.00	12.00	12.00
Sunflower meal, %	13.01	14.99	12.04
Sunflower oil, %	1.00	1.00	1.00
Rape cake, %	10.00	-	-
Hemp cake, %	-	10.00	-
Camelina cake, %	-	-	10.00
Brewers’ yeast, %	5.00	5.00	5.00
Oyster shell, %	0.50	0.50	0.50
Fodder chalk %	1.25	1.44	1.27
Premix “Calvet”, %	1.00	1.00	1.00
Fodder salt, %	0.10	0.10	0.10
Monocalcium phosphate, %	0.78	0.50	0.76
Calculated nutritional value of feed mixture			
Dry matter, kg/kg	0.87	0.87	0.87
Metabolizable energy (ME), MJ/kg	11.07	11.26	11.19
Crude protein, g/kg	190.04	190.04	190.05
Lysine, g/kg	8.95	8.41	8.68
Methionine, g/kg	6.24	6.41	6.52
Fat, g/kg	43.59	37.59	38.15
Fiber, g/kg	58.14	76.17	58.54
Ca, g/kg	12.40	12.40	12.41
P, g/kg	7.30	7.30	7.29

**Table 2:** The proportions (%) of main fatty acid groups and their ratio in the fat of quail feed.

Groups and Ratio of Fatty Acids	Feed Group		
	Rapeseed	Hemp	Camelina
SFAs	12.68	12.40	13.29
MUFAs	19.23	17.76	21.08
PUFAs	68.09	69.84	65.63
n-6 PUFAs	64.24	65.06	51.99
n-3 PUFAs	3.85	4.78	13.64
ALA (C18:3n-3)	3.82	4.77	13.25
n-6/n-3 PUFAs	16.69	13.61	3.81

**Table 3:** Effects of diet on egg and yolk weight in different quail groups.

Variables	Feeding Group			SED	*p*-Value
	Rapeseed *n* = 15	Hemp *n* = 15	Camelina *n* = 15		
Weight of egg, g	10.40 ^A^	9.20 ^a^	10.14	0.498	0.047
Weight of yolk, g	3.96	3.66	4.03	0.372	0.567
Yolk, %	37.99	39.52	39.72	3.038	0.825

The differences between the means of groups in the rows with different superscripts differ at ^A-a^ *p* ˂ 0.05; SED—standard error of difference.

**Table 4:** Effects of diet on pH and colour of egg yolk and albumen in different quail groups.

Variables		Feeding Group			SED	*p*-Value
		Rapeseed *n* = 15	Hemp *n* = 15	Camelina *n* = 15		
pH	Yolk	7.10	7.19	7.07	0.087	0.365
	Albumen	9.38 ^C,B^	9.17 ^c^	9.24 ^b^	0.048	0.000
Colour L*	Yolk	81.17 ^B^	78.74 ^b,A^	80.48 ^a^	0.690	0.005
	Albumen	65.68 ^B^	64.35	63.15 ^b^	0.639	0.002
a*	Yolk	−7.88 ^C^	−5.77 ^c^	−8.64 ^C^	0.450	0.000
	Albumen	−5.57	−5.98	−4.94	1.028	0.601
b*	Yolk	45.45	47.16	45.45	1.736	0.531
	Albumen	6.31 ^C^	8.59 ^c^	9.21 ^c^	0.463	0.000
C	Yolk	46.24	47.52	46.37	1.710	0.717
	Albumen	8.43 ^C^	10.47 ^c^	11.10 ^c^	0.424	0.000
h	Yolk	99.77 ^C^	96.98 ^c^	100.75 ^C^	0.514	0.000
	Albumen	131.97 ^C^	125.01 ^c^	124.07 ^c^	1.401	0.000

SED—standard error of difference; L*—lightness; a*—redness/greenish; b*—yellowness; C—chroma; h—hue angle; *p* values of GLM post hoc LSD tests for the groups were significantly different at ^A-a^ *p* ˂ 0.05; ^B-b^ *p* ˂ 0.01; ^C-c^ *p* ˂ 0.001.

**Table 5:** Effects of diet on egg yolk proximate composition and cholesterol content in different quail groups.

Variables	Feeding Group			SED	*p*-Value
	Rapeseed *n* = 15	Hemp *n* = 15	Camelina *n* = 15		
Dry matter, %	49.50 ^B^	48.17 ^b^	49.54 ^B^	0.410	0.003
Protein, %	14.05	13.07 ^B^	14.66 ^b^	0.557	0.026
Lipids, %	31.38	30.42	31.32	2.085	0.122
Ash, %	1.86	1.81	1.78	0.062	0.436
Cholesterol, mg/g	10.61 ^A,B^	12.94 ^a^	13.18 ^b^	0.853	0.010

SED—standard error of difference; *p* values of GLM post hoc LSD tests for group were significantly different at ^A-a^ *p* ˂ 0.05; ^B-b^ *p* ˂ 0.01.

**Table 6:** Effects of diet on fatty acid composition (%) in yolk lipids of different quail groups.

Fatty Acids	Feeding group			SED	*p*-Value
	Rapeseed *n* = 15	Hemp *n* = 15	Camelina *n* = 15		
C14:0	0.38 ^B^	0.48 ^b^	0.45 ^b^	0.039	0.027
C15:0	0.01	0.01	0.02	0.008	0.373
C16:0	22.78 ^A,B^	25.28 ^b^	24.73 ^a^	0.832	0.014
C17:0	0.08	0.10	0.11	0.013	0.110
C18:0	8.22	8.17	8.40	0.396	0.826
C22:0	0.12	0.08	0.07	0.032	0.213
SFAs	31.58 ^B^	34.11 ^b^	33.77 ^B^	0.789	0.007
C14:1n-7	0.08 ^B^	0.14 ^b^	0.10	0.021	0.030
C16:1n-9	0.87 ^C,A^	0.54 ^c^	0.69 ^a^	0.075	0.001
C16:1n-7	4.21 ^B^	6.02 ^b^	5.11	0.627	0.026
C17:1n-9	0.06 ^A^	0.07	0.08 ^a^	0.009	0.042
C18:1n-9trans	0.14	0.13	0.13	0.010	0.307
C18:1n-9	44.99 ^C^	38.83 ^c,B^	42.93 ^b^	1.432	0.001
C18:1n-7	2.59	2.40	2.37	0.186	0.452
C20:1n-9	0.15 ^B^	0.15 ^B^	0.20 ^b^	0.018	0.009
MUFAs	53.08 ^B^	48.28 ^b,A^	51.62 ^a^	1.220	0.002
C18:2 n-6t,c	0.08	0.07	0.07	0.006	0.192
C18:2 n-6	10.92	12.67 ^B^	9.76 ^b^	0.991	0.023
C18:3 n-6	0.20 ^C^	0.27 ^c^	0.17 ^C^	0.016	0.000
C18:3 n-3	0.23 ^C,B^	0.38 ^C,b^	0.64 ^c^	0.039	0.000
C20:2 n-6	0.05 ^A^	0.08 ^a^	0.07	0.010	0.036
C20:3 n-6	0.15	0.21	0.17	0.027	0.095
C20:4 n-6	2.63 ^C^	2.49 ^C^	1.94 ^c^	0.126	0.000
C20:5 n-3	0.00	0.02	0.09	0.010	0.000
C22:4 n-6	0.10 ^B,A^	0.14 ^b,C^	0.07 ^a,c^	0.013	0.000
C22:5 n-3	0.10 ^C,B^	0.15 ^c^	0.21 ^c,b^	0.016	0.000
C22:6 n-3	0.52 ^A,C^	0.68 ^a,C^	1.10 ^c^	0.058	0.000
PUFAs	14.98	17.15 ^A^	14.28 ^a^	1.071	0.032
TFAs	0.22	0.20	0.19	0.012	0.137
UFA	0.39 ^A^	0.47 ^a,B^	0.34 ^b^	0.041	0.011

SED—standard error of difference; SFAs—all identified saturated fatty acids; MUFAs—identified monounsaturated fatty acids; PUFAs—all identified polyunsaturated fatty acids; TFAs—all identified trans fatty acids; UFAs—unidentified fatty acids; *p* values of GLM post hoc LSD tests for the groups were significantly different at ^A-a^ *p* ˂ 0.05; ^B-b^ *p* ˂ 0.01; ^C-c^ *p* ˂ 0.001.

**Table 7:** Effects of diets on fatty acid ratios and lipid quality indices in egg yolk lipids of different quail groups.

Variables	Feeding Group			SED	*p*-Value
	Rapeseed *n* = 15	Hemp *n* = 15	Camelina *n* = 15		
PUFAs/SFAs	0.47	0.51 ^A^	0.42 ^a^	0.038	0.105
n-6/n-3	16.59 ^C^	13.09 ^c,C’^	6.04 ^c,c’^	0.655	0.001
AI	0.36 ^B,A^	0.42 ^b^	0.40 ^a^	0.018	0.008
TI	0.87 ^A^	0.95 ^a^	0.88 ^A^	0.032	0.038
h/H	2.72 ^B,A^	2.28 ^b^	2.38 ^a^	0.126	0.004
PI	29.24 ^A^	32.71 ^a^	31.78	1.440	0.060

PUFAs/SFAs—ratio of PUFAs to SFAs; n-6/n-3—ratio of n-6 PUFAs to n-3 PUFAs, AI—atherogenic index, TI—thrombogenic index, h/H—hypocholesterolemic/hypercholesterolemic ratio, PI—peroxidisability index. *p* values of GLM post hoc LSD tests for the groups were significantly different at ^A-a^ *p* ˂ 0.05; ^B-b^ *p* ˂ 0.01; ^C-c;C’-c’^ *p* ˂ 0.001.

## Data Availability

Not applicable.

## Supplementary Materials

The following supporting information can be downloaded at: https://www.mdpi.com/article/10.3390/ani12223110/s1, Table S1: Composition of premix “Calvet“.
