# Supplementary material for: Effects of Dietary Rapeseed (Brassica napus), Hemp (Cannabis sativa) and Camelina (Camelina sativa) Seed Cakes Supplementation on Yolk and Albumen Colour and Nutritional Value of Yolk Lipids in Estonian Quail Eggs
Source: Animals (Basel). 2022 Nov 10;12(22):3110. doi: 10.3390/ani12223110 (PMC9686490; doi:10.3390/ani12223110)
Supplement: Supplementary file 1 [file animals-12-03110-s001.zip › animals-1921549-supplementary.pdf]

Table S1. Composition of premix „Calvet" (producer: Dolfos, Poland)

	Units	Amount
Calcium	g/kg	195.0
Phosphorus	g/kg	6.30
Sodium	g/kg	0.34
Iron	mg/kg	153.60
Zinc	mg/kg	44.10
Vitamin A	IU	640.00
Vitamin E	mg	3.00
Vitamin B1	mg	6.60
Vitamin B2	mg	19.90
Vitamin B6	mg	13.20
Vitamin B12	mg	0.02
Biotin	mg	0.88
Nicotinic acid	mg	226.40
Pantothenic acid	mg	17.70
Folic acid	mg	8.80
Lysine	g/kg	17.90
Methionine	g/kg	3.80
Cystine	g/kg	1.80
Threonine	g/kg	11.80
Tryptophan	g/kg	2.20
Isoleucine	g/kg	11.00
Tyrosine	g/kg	21.00
Phenylalanine	g/kg	23.40
Valine	g/kg	11.70
Choline	g/kg	2.30
Histidine	g/kg	8.60
Arginine	g/kg	12.90
Proline	g/kg	4.50
Asparagine	g/kg	21.00
Leucine	g/kg	15.00
